# Three-dimensional printing as an aid to airway evaluation after tracheotomy in a patient with laryngeal carcinoma

**DOI:** 10.1186/s12871-015-0170-1

**Published:** 2016-01-19

**Authors:** Bin Han, Yajie Liu, Xiaoqing Zhang, Jun Wang

**Affiliations:** Department of Anesthesiology, Peking University Third Hospital, No. 49 North Garden Street, Haidian District, Beijing, 100191 China

**Keywords:** 3D printing, Anesthesia, Difficult airway

## Abstract

**Background:**

Difficult airway may result in significant morbidity and mortality. Proficient airway evaluation, therefore, is one of the key elements in the safe conduct of anesthesia. A three-dimensional (3D) printing model was recently introduced for medical application. 3D printing is a fast, convenient, and relatively affordable technique. We present a case in which a 3D-printed airway model was successfully used for airway evaluation.

**Case presentation:**

A 77-year-old man who had previously undergone total laryngectomy was scheduled for resection of a pelvic mass. The condition of his airway, however, complicated the procedure. Routine methods to evaluate his airway were not suitable. Therefore, the patient’s computed tomography imaging data were used to generate stereolithography files and then to print out 3D models of his trachea. These 3D models enhanced our understanding of his tracheal morphology. They helped us devise a preanesthesia plan and effectively execute it without complications.

**Conclusion:**

3D printing models allow better understanding of morphological changes in the airway and aid preanesthesia planning. The successful outcome of our case suggests 3D printing is a potent tool for evaluating difficult and more widespread use is encouraged.

## Background

Failures in tracheal intubation caused by difficult airways frequently result in lethal anesthesia-related complications [1]. It has been reported that many anesthesia-related deaths are attributed to improper handling of a difficult airway [2]. The airway structures in patients with laryngeal carcinoma undergo considerable change after surgery, and various lesions may ensue, including granulomas, tracheomalacia, and esophagotracheal fistula, among others [3, 4]. Also, the established artificial airway may weaken the nonspecific respiratory defense capacities, which would increase the risk of chronic lung disease. Hence, airway evaluation is one of the key elements in anesthetic management of such patients.

Rapid prototyping is a process of producing a 3D model layer by layer based on computer-aided design (CAD) data or images that precisely represent the anatomical details of a defect [5]. As it is becoming better known, 3D printing is becoming increasingly popular worldwide. Having been used for decades in industrial design, it has recently shown potential in medical applications [6], especially for implant designing and preoperative planning [7, 8]. As far as we are aware, however, this technique has not been used to assess difficult airways for preanesthesia evaluation. In this report, we present a case in which a new method of producing a 3D-printed airway model was used to evaluate a patient’s airway before anesthesia induction.

## Case presentation

**Fig. 1:**
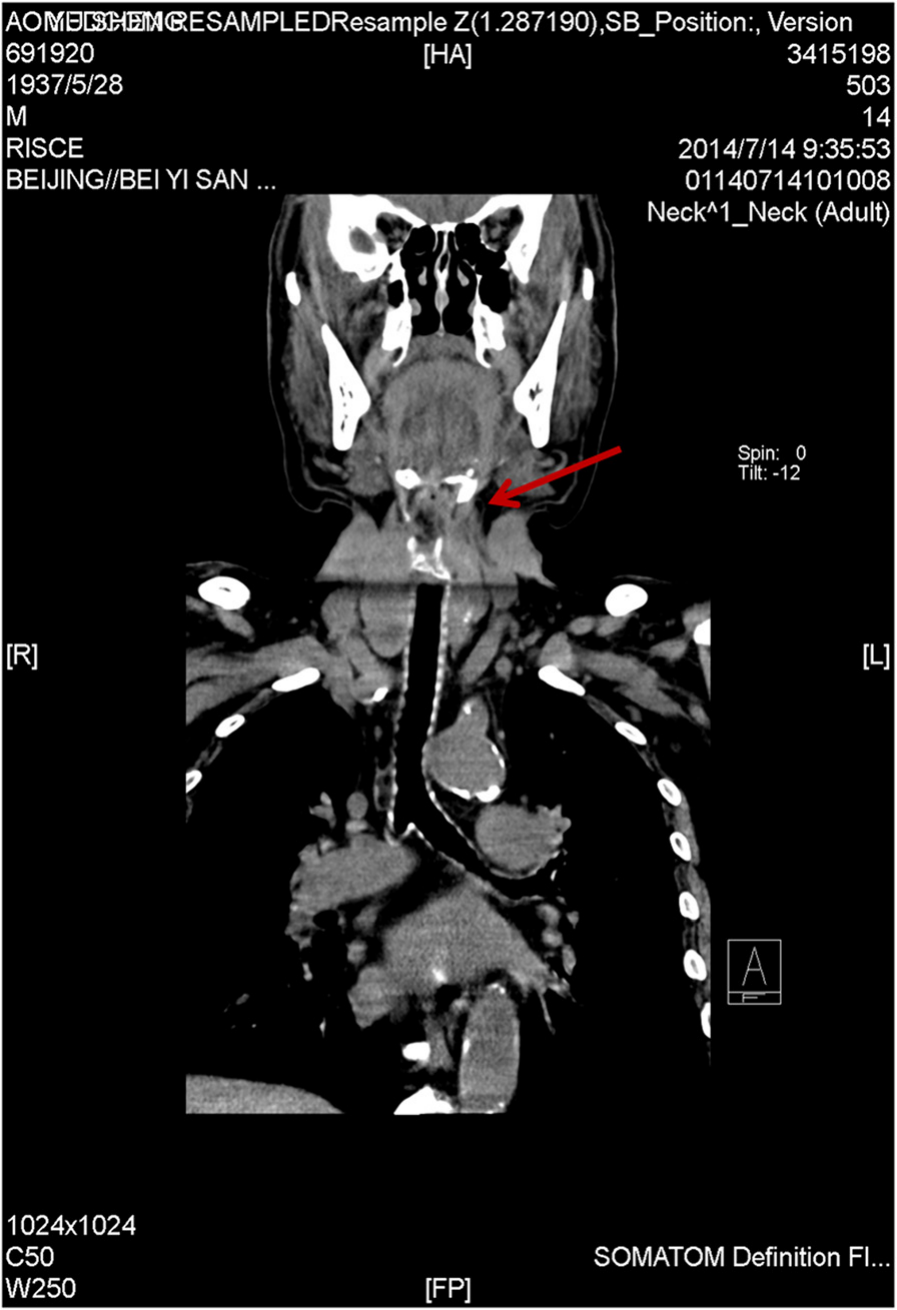
Computed tomography of the neck

A 77-year-old man diagnosed with a pelvic mass was admitted to the operating room for resection of the mass. The patient had a 15-year history of diabetes as well as nephropathy during a period of uremia, hypertension with irregular use of an antihypertensive drug, laryngeal carcinoma for 37 years, lacunar infarct, and cataract. He had undergone cataract surgery and total laryngectomy previously, with the tracheostomy cannula removed 3 months ago. Physical examination revealed that a fistula had formed at the site of the tracheotomy incision. Computed tomography (CT) of the neck (Fig. 1) revealed that the soft tissue around the airway was asymmetrical below the hyoid bone, with thickening on the left side. The preanesthesia evaluation indicated American Anesthesiologists Association (ASA) category III, and we might confront two possible difficulties during the intubation: 1) the patients had removed tracheostomy cannula for 3 months, whether the stoma retracted? 2) Whether there existed tracheostenosis below the stoma? These suspected predictors indicated the infra-hyoid difficult airway.

**Fig. 2:**
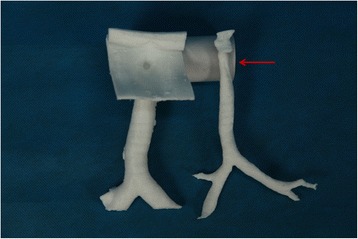
3D models of the inside and outside tracheal diameters

The CT examination of the trachea was performed using a CT scanner (SOMATOM Definition flash; Siemens, Erlangen, Germany). Scanning parameters were as follows: slice thickness 3 mm, intervals 3 mm, 120 kV, and 252 mAs. Scan acquisitions were imported into a software program (Mimics 15.0; Mimics, Gilching, Germany). The tracheal area was selected to set the threshold using the thresholding function. The sliced images were then created using the region growing function and were ultimately reconstructed by calculation using the 3D function. The 3D reconstructed image was then exported in standard tessellation language (STL) format. These images were directly loaded into the 3D printer (MakerBot Replicaor 2; MakerBot, Brooklyn, NY USA) to enable production of 3D polylactic acid models of the inside and outside tracheal diameters (Fig. 2). Almost 10 h was needed to prepare the STL file and to print the models. These models were white and moderately hard, which met our needs.

With the aid of the 3D model, we found slight scar retraction surrounding the stoma created by the tracheostomy and slight stenosis below the stoma in the trachea as suspected. With accurate data for the tracheal inner diameter, we created an anesthesia plan. We then practiced every intubation step on the 3D model, finally choosing a No. 8 tracheal catheter and No. 8 tracheostomy cannula. Because the patient would be transferred to the intensive care unit (ICU) postoperatively, where he would be given assisted mechanical ventilation and respiratory therapy, we decided to use the No. 8 tracheostomy cannula, which would be well tolerated and allow easy fixation.

In the operating room (OR), routine monitoring was performed with electrocardiography, noninvasive blood pressure, and pulse oximetry. The initial arterial blood pressure was 152/79 mmHg with a heart rate of 78 beats/min. Sinus rhythm was present, and the peripheral oxygen saturation (SpO_2_) was 98 %. A radial arterial line was placed after the Allen test indicated it was safe. The patient was premedicated with intravenous atropine (0.5 mg) to reduce airway secretions. Topical anesthesia was achieved with 3 ml of 2 % lidocaine. A fiberoptic bronchoscope was then inserted though the stoma to reevaluate the airway. An intravenous propofol bolus of 30 mg was given, after which the No. 8 tracheostomy cannula was inserted. An otolaryngologist was present in the OR and was ready to enlarge the stoma if it was difficult to establish the artificial airway. Immediately after intubation, intravenous propofol and atracurium were administered to induce general anesthesia and to establish mechanical ventilation. Maintenance was provided with sevofluorane 2 %, atracurium, and remifentanil. Urologists performed the tumor resection and partial cystectomy. At the end of the procedure, the patient was transferred to the ICU with the tracheostomy cannula in place. Three days later, the patient was transferred to the inpatient floor with no respiratory or hemodynamic impairment.

## Discussion

According to clinical experience, tracheostomy is often associated with complications, such as granulomas, tracheomalacia, and tracheostenosis. And tracheostenosis can be seen in the late stage of tracheostomy, which is due to: 1) the contact with the tip of the rigid intubation tubes or tracheotomy tube, 2) injury caused by high cuff pressure leads to an end of the infusion of capillaries in the tracheal mucosa leading to ischemia. Healing by fibrosis leads to tracheal stenosis [9]. Besides, stoma is deprived of supports by tracheal cartilage may cause stoma stenosis. In addition, stoma retraction is one of the challenges that the anesthesiologist often confronts.

In patients who present with a difficult airway, the preanesthesia evaluation is crucial to anesthesia management and patients’ outcomes [10]. There are various methods for evaluating difficult airways, including the Mallampati classification, interincisor distance, thyromental distance, and range of motion of the head and neck [11]. These methods, however, were not suitable for this patient as they apply to routine intubation with a laryngoscopic view.

Imaging techniques such as CT and plain radiography are important aids for visualizing anatomy and pathology [12]. In the present case, the 3D model was particularly important for aiding procedure planning and choosing the proper cannula location and size. We shortened the intubation time, increased intubation success rate, and avoided injuries caused by repeated intubation.

We used, for the first time, the 3D printout technique in a patient with laryngeal carcinoma who had undergone previous tracheotomy to present a new method of evaluating the difficult airway. The benefits of evaluating the airway with 3D models include the following: (1) 3D models are easy to interpret by the anesthesiologist and can better demonstrate tracheal anatomical structures and their relations with surrounding tissues. (2) 3D models enhance the anesthesiologist’s understanding of the morphology, which may have been significantly altered by the underlying pathology. It could also help facilitate communication with team members to create a better preanesthesia plan. (3) 3D models with direct vision could enable the anesthesiologist to assess risk factors, adjust the plan, and provide manual accuracy. Accordingly, it would help shorten the intubation time and reduce injuries in patients. (4) Subsequent benefits may include medical education for interns and better understanding for patients.

The 3D printout technique also has limitations. It does not recognize color variations in the tracheal mucosa and thus cannot distinguish inflammation of the airway wall. Under these circumstances, visual techniques such as fiberoptic bronchoscopy can be helpful as a complement to the 3D printout technique.

## Conclusion

In conclusion, 3D printing was a groundbreaking technique and has matured. The successful outcomes of our case suggest the great practicability of preanesthesia planning, and more widespread use is encouraged. In the future, this technology could be better used not only to evaluate the difficult airway but also create artificial tracheal tubes for specific patients, thereby realizing individualized anesthesia.

## Consent

Written informed consent was obtained from the patient for publication of this case report. A copy of the written consent is available for review by the Editor of this journal.

## Abbreviations

3D: three-dimensional
ASA: American Anesthesiologists Association
CAD: computer-aided design
CT: computed tomography
ICU: intensive care unit
OR: operating room
SpO_2_: peripheral oxygen saturation
STL: standard tessellation language
